# Sudden cardiac death in young adults and the role of antipsychotic drugs: a multicenter autopsy study

**DOI:** 10.1007/s12024-025-01096-3

**Published:** 2025-10-21

**Authors:** Massimiliano Esposito, Pietro Zuccarello, Francesco Sessa, Emanuele Capasso, Arianna Giorgetti, Mauro Pesaresi, Monica Salerno, Nunziata Barbera, Cristoforo Pomara

**Affiliations:** 1 Faculty of Medicine and Surgery, “Kore” University of Enna, Enna, 94100 Italy; 2 Department of Psychology and Health Sciences, Pegaso Telematic University, Naples, 80143 Italy; 3 Department of Medical, Surgical and Advanced Technologies “G.F. Ingrassia”, University of Catania, Catania, 95121 Italy; 4 Department of Advanced Biomedical Science-Legal Medicine Section, University of Naples “Federico II”, Naples, 80131 Italy; 5 Unit of Legal Medicine, Department of Medical and Surgical Sciences, University of Bologna, Via Irnerio 49, Bologna, 40126 Italy; 6 Section of Legal Medicine, Department of Biomedical Science and Public Health, Marche Polytechnic University, Ancona, 60126 Italy

**Keywords:** Sudden cardiac death, Antipsychotic drugs, Autopsy study, Histopathological findings

## Abstract

Sudden cardiac death (SCD) in patients with schizophrenia treated with antipsychotic drugs (APDs) has become a significant concern, with increasing incidence, particularly among young adults. This multicenter study aimed to investigate the cardiac effects of APDs in a cohort of patients who died from SCD. A retrospective analysis was conducted on seven schizophrenic patients, aged 25 to 39 years, who died from SCD while receiving antipsychotic therapy. Patients with cardiovascular risk factors, substance abuse at the time of death, or advanced age were excluded. Autopsies focused on cardiac tissue, and histological and toxicological analyses were performed. The findings revealed structural cardiac changes, including myocardial fibrosis, myofiber disruption, and contraction band necrosis—features consistent with arrhythmic SCD. Toxicological results confirmed APD use in all cases, with polypharmacy patterns commonly observed. The study suggests that APDs can induce significant cardiac alterations that may predispose patients to SCD, even in the absence of pre-existing cardiac disease. These findings underscore the need for careful cardiac monitoring in patients with schizophrenia treated with APDs, particularly those without traditional cardiovascular risk factors. Further research, including molecular autopsy and genetic analysis, is necessary to better understand genetic predispositions and the role of APDs in SCD.

## Introduction

Antipsychotic drugs (APDs) are commonly prescribed for the treatment of schizophrenia and other serious psychiatric disorders [1]. Sudden cardiac death (SCD) can be a side effect of certain APDs in psychiatric patients, leading to the withdrawal of some drug classes [2]. Both typical and atypical APDs have been reported to significantly increase the risk of SCD. Recently, medical devices—including those incorporating artificial intelligence (AI)—have been developed to detect and report irregular heart rhythms or arrhythmias, aiming to prevent SCD [1–3].

A case-crossover study using data from the Taiwan National Research Database [4] applied conditional logistic regression models and found that APD use was associated with a 1.53-fold increased risk of ventricular fibrillation-induced SCD, particularly among short-term users. Hennessy et al. [5] identified thioridazine as one of the APDs with the highest risk of adverse cardiovascular events. Despite decades of research, the relationship between APDs and SCD remains unclear. This is partly due to the presence of multiple concomitant risk factors in patients with schizophrenia, including genetic predisposition, smoking, and the use of other medications or substances, which complicate the assessment of APD-specific risks [5, 6]. A systematic review [7] analyzed the association between opioid overdose and APD use. Quetiapine, in particular, is frequently found in overdose cases due to its enhancement of opioid-induced sedation, leading to respiratory depression, hypotension, and QT interval prolongation. A pharmacokinetic interaction between quetiapine and methadone may exacerbate these effects. Additionally, a recent meta-analysis [8] revealed an increased risk of cardiovascular complications and SCD associated with quetiapine, olanzapine, risperidone, haloperidol, and thioridazine, highlighting the importance of considering these risks when prescribing APDs.

A recent study examined nine schizophrenia patients who died from SCD during hospitalization and underwent autopsy [9]. These patients, both male and female, had a mean duration of schizophrenia of 6.83 ± 3.75 years. Among them, four deaths were attributed to acute coronary syndrome (ACS), one to myocarditis, one to cardiomyopathy, one to pulmonary thromboembolism, and in two cases, no specific cause of death was identified. The authors noted that postmortem studies in this population are rare and that the patients are generally young adults. These individuals often present with additional risk factors, such as unhealthy lifestyles and obesity. The effectiveness of ECG monitoring in preventing SCD remains uncertain, as six of the nine subjects were under continuous ECG monitoring at the time of death [9].

This article presents the first forensic multicenter study focusing on cardiac changes induced by APDs in patients with schizophrenia who were chronically treated with these drugs. While previous studies are primarily retrospective and based on database analyses with limited autopsy details, the current study specifically examines cardiac alterations through comprehensive autopsy investigations. This manuscript contributes to both forensic and broader scientific communities by providing insights into the primary cardiac alterations induced by APDs, as observed through macroscopic and microscopic examinations. It also enhances understanding of how these drugs may lead to SCD in young adults.

## Materials and methods

A multicenter retrospective analysis was conducted on autopsies performed between 2020 and 2024 at the Departments of Forensic Pathology in Enna, Catania, Naples, and Bologna. The study included patients with schizophrenia who had been chronically treated with antipsychotic drugs (APDs) and underwent complete autopsy investigations. Blood samples were collected from the femoral vein, and hair samples were also obtained. Cadavers were transported to the morgue and refrigerated at − 4 °C. Autopsies were performed within 36 h postmortem. Histological and toxicological examinations were conducted, and the cause of death was determined as sudden cardiac death (SCD) according to ESC guidelines [10, 11].

### Study population

A retrospective analysis was conducted on subjects who died of SCD. None of the patients were hospitalized and were treated with noradrenergic drugs. The inclusion criteria were as follows: young adults aged 18–39 years [12], death attributed to SCD as determined through complete autopsy, a history of psychosis treated with APDs for more than 3 years, a normal body mass index (BMI) ranging from 18.5 to 24.9 kg/m², absence of congenital or acquired cardiac diseases, no history of acute diseases, and toxicological findings consistent with therapeutic APD levels.

Exclusion criteria included overweight or obesity, use of narcotics at the time of death, age over 40 years, use of other drugs in addition to APDs that that could affect cardiac tissue, decomposed bodies, death from a cause other than SCD, and autopsies performed more than 48 h postmortem. After applying the inclusion and exclusion criteria, seven cases were included.

The inclusion and exclusion criteria were designed to ensure a more homogeneous sample and to exclusively assess the effects of APDs on myocardial tissue. Excluding subjects under 18 years of age was intended to minimize potential bias from undiagnosed genetic conditions, though such conditions can also manifest in adulthood, while excluding individuals over 40 years of age helped avoid the confounding influence of age-related pathologies. Additionally, individuals with other cardiac risk factors, such as obesity, congenital or acquired cardiac diseases, or the use of other drugs that affect the heart, were excluded. Finally, to minimize the impact of postmortem changes in blood concentrations of APDs, autopsies and toxicological analyses were conducted within two days of death, and the bodies were stored in a cold room at −2 °C [13].

## Autopsy procedure

Autopsies were performed in accordance with SCD guidelines [14, 15]. Prior to the autopsy, an external examination of the body was conducted to exclude external causes of death (e.g., signs of violence, self-harm). Height, weight, and BMI were recorded. A total body CT scan was performed using a helical 16-slice CT scanner (Philips CT Brilliance 16), and the scan results were interpreted by a forensic radiologist. The autopsy was performed in sections, with the heart being weighed and measured, then extracted in its entirety and fixed in 10% buffered formalin at pH 7 [16]. After 20 days, the fully formalin-fixed heart was studied, and a careful examination of the coronary arteries, cardiac walls, valves, and great vessels was conducted. The thicknesses of the heart chambers were measured. Eleven heart samples were taken, including one sample from the apex, one from the anterior left ventricle, one from the lateral left ventricle, one from the posterior left ventricle, one from the anterior septum, one from the posterior septum, one from the right ventricle, and one from each heart valve. Additionally, four coronary artery samples were collected (common trunk, anterior descending artery, circumflex artery, and right coronary artery) [16].

## Histological analysis

Histopathological examination was performed using hematoxylin-eosin (H&E) staining. Heart specimens were fixed in 10% buffered formalin as described above, then washed overnight, dehydrated in graded ethanol, cleared in xylene, and embedded in paraffin. The paraffin blocks were sectioned to a thickness of 4 μm using a microtome, and the sections were mounted on silane-coated slides (Dako, Glostrup, Denmark) and stored at room temperature. H&E staining was performed. A Zeiss Axioplan optical microscope (Carl Zeiss, Oberkochen, Germany) was used to observe the histological preparations, and photographs were captured using a Zeiss AxioCam MRc5 digital camera (Carl Zeiss, Oberkochen, Germany).

## Toxicological analysis

Blood samples were collected immediately after death and prior to the autopsy from the femoral vein [17]. To minimize changes in postmortem blood concentrations of APDs, autopsy and toxicological analyses were performed within two days of death, and the bodies were stored in a cold room at −2 °C [13, 18].

## Blood extraction

For each case, a 0.1 ml aliquot of peripheral blood was spiked with a mixture of bupivacaine (1,000 ng/ml) and clozapine (200 ng/ml) as internal standards. Subsequently, 0.1 ml of 0.1 N sulfuric acid was added to the sample. After manual mixing, 0.3 ml of acetonitrile was added to induce protein precipitation. Following centrifugation at 8,000 rpm for 2 min, the supernatant was transferred to a vial for subsequent analysis.

### Hair extraction

For each case, a 1-gram hair sample (3 cm from the scalp) was collected during the autopsy. After decontamination by washing 3 For each case, a 1-gram hair sample (3 cm from the scalp) was collected during the autopsy. After decontaminating the hair by washing it three times with 5 ml of dichloromethane, it was finely chopped. An aliquot of 100 mg of hair was placed into a glass flask, and an appropriate mix of internal standards (bupivacaine 5 ng/mg) was added, along with 1 ml of 0.1 N HCl. The sample was incubated for 24 h at 45 °C. The aqueous phase was then extracted twice with 2 ml of a chloroform-isopropyl alcohol mixture (80:20 v/v), first at pH 7 (by adding 1 M phosphate buffer) and then at pH 11 (by adding 1 N sodium hydroxide). The two extracts were combined, the solvent was evaporated under a nitrogen stream, and the dry residue was dissolved in 30 µl of acetonitrile.

#### LC-MS analysis

The extracts from blood and hair samples were analyzed using Ultra-High-Pressure Liquid Chromatography with Electro-Spray Ionization (UHPLC-ESI) and detection by Triple Quadrupole Mass Spectrometry. An Acquity UHPLC-ESI-TQD system from Waters was used, equipped with an Acquity UPLC^®^ T3 C18 1.8 μm – 2.1 × 150 mm column and a mobile phase consisting of water and acetonitrile, both containing 0.1% formic acid, with the percentage composition varying during the run. The sample volume injected into the chromatograph was 10 µl.

#### Ethical approval

All procedures were performed in accordance with the 1964 Helsinki Declaration and its subsequent amendments. The study was approved by the Ethics Committee (code 05/CEL). In order to maintain confidentiality, each cadaver was assigned an alphanumeric code and subsequently processed in an internal database.

## Results

### Study population

The study population consisted of 6 males and 1 female, with ages ranging from 25 to 39 years, and a median age of 29 ± 4.71 SD. The duration of psychosis and treatment with antipsychotic drugs (APDs) ranged from 5 to 10 years, with a median of 5 years. The body mass index (BMI) of the participants was within the normal weight range, with values ranging from 19 to 23, and a mean of 22 ± 1.6 SD. None of the patients had cardiovascular risk factors or a history of substance abuse at the time of death.

### Autopsy procedure

At autopsy, the weight of the formalin-fixed hearts ranged from 332 g to 520 g, with a mean of 350 ± 78 SD. 

**Fig. 1:**
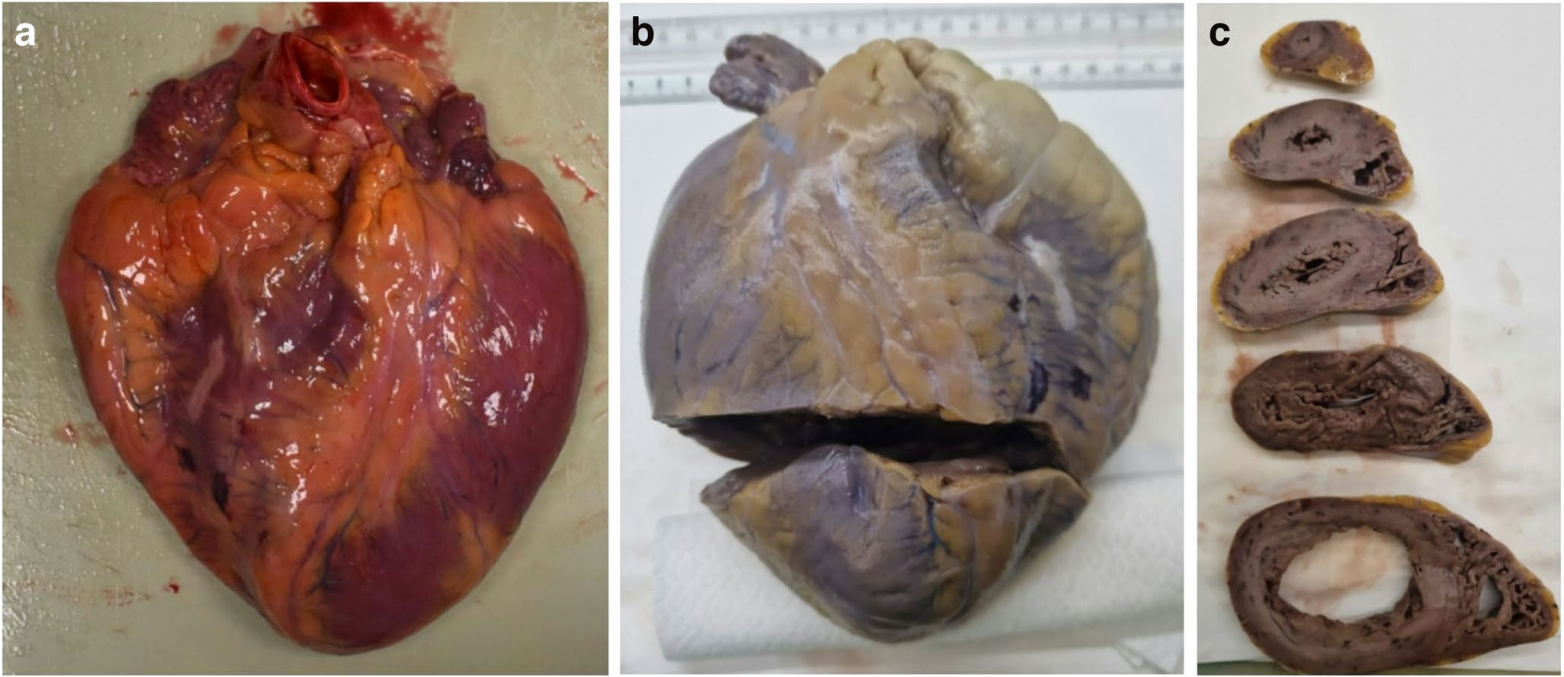
Hemorrhagic petechiae and the whitish area in the right ventricle (**a**-**b**). Sections of the heart after fixation in 10% formalin (**c**)

Case 1 displayed petechial hemorrhages in the right ventricle with a white ischemic area, while Case 2 demonstrated left ventricular myocardial hypertrophy Case 3 exhibited petechial hemorrhages in the right ventricle with a white ischemic area, mild thickening of the parietal endocardium, and non-hemodynamically significant stenosis of the circumflex coronary artery (Fig. 1). Case 4 showed dilation of the ventricular chambers. Cases 5, 6, and 7 showed no pathological signs upon macroscopic examination.

### Histological analysis

**Fig. 2:**
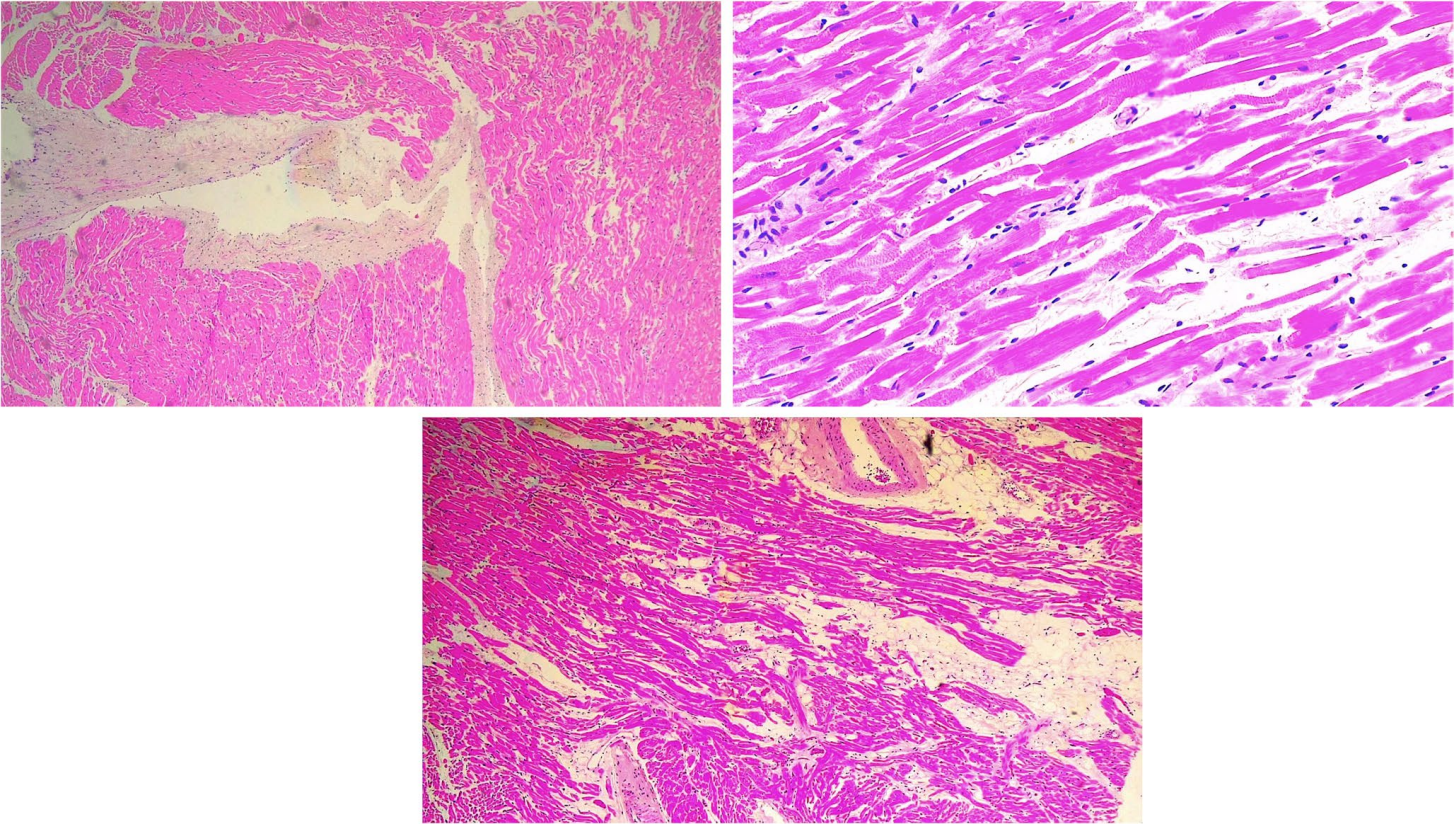
Diffuse areas of connective tissue, contraction band necrosis, perivascular fibrosis, leukocytic infiltration of myofibers, and myofiber break-up

Microscopic examination of the heart tissue stained with hematoxylin-eosin (H&E) revealed myofiber break-up of cardiomyocytes, interstitial and perivascular fibrosis of the myocardial tissue, contraction band necrosis, square nuclei, and waviness in all cases. Leukocytic infiltration of myofibers was observed in Cases 1, 3, and 5. Coronary atherosclerosis was present in Cases 2, 4, and 6. Figure 2 summarizes some of the main histological findings. In Case n.3 H&E examination revealed significant evidence of myocardial damage, including myofiber break-up of cardiomyocytes and prominent interstitial and perivascular fibrosis. The presence of contraction band necrosis points to a sudden, hypercontractile event preceding death. We also observed wavy fibers and leukocytic infiltration, indicating an inflammatory response and potential cellular damage. The cardiomyocytes exhibited square nuclei, a feature often associated with cellular hypertrophy and chronic stress.

### Toxicological analysis

Toxicological analysis revealed the presence of APDs in both blood and hair in all cases, indicating that the patients had been taking APDs both before death and in the preceding months. In 4 cases (Cases 1, 3, 4, and 6), the antipsychotic drugs detected in blood were the same as those found in hair, while in the remaining 3 cases (Cases 2, 5, and 7), the drugs identified in hair were different from those found in blood. Blood toxicology most commonly detected haloperidol, promazine, aripiprazole, and clozapine (each detected in 3 cases), followed by quetiapine (2 cases) and clomipramine, paliperidone, and pimozide (1 case each). Hair toxicology revealed quetiapine, clozapine, and promazine (each detected in 3 cases), followed by haloperidol and aripiprazole (2 cases) and pimozide and clomipramine (1 case each). The results of the toxicological analysis of blood and hair suggest that most patients were undergoing polypharmacy with APDs both acutely and chronically. Table 1 summarizes all the results of the study.

**Table 1:** Summary of the study results

Age	Gender	Psychosis history (years)	BMI	Heart weight (gr)	Macroscopic findings of the heart	Histological analysis	Toxicological analysis (blood)	Toxicological analysis (hair)
25	M	5	22	353	Petechiae hemorrhages right ventricle with a white ischemic area	Myofiberbreak-up of cardiomyocytes. Interstitial and perivascular fibrosis of the myocardial tissue. Contraction band necrosis. Square nuclei. Myocell infiltration of leukocytes. Waviness	Quetiapine	Quetiapine
28	M	6	21.5	342	Left ventricular myocardial hypertrophy	Myofiberbreak-up of cardiomyocytes. Interstitial and perivascular fibrosis of the myocardial tissue. Contraction band necrosis. Square nuclei. Waviness. Coronary atherosclerosis not hemodynamically significant (< 50%).	Haloperidol, Clotiapine, Paliperidone, Promazine	Haloperidol, Clotiapine, Promazine
29	M	10	23	520	Petechiae hemorrhages right ventricle with a white ischemic area. Areas of mild thickening of the parietal endocardium. Non-hemodynamically significant stenosis of the circumflex coronary artery.	Myofiberbreak-up of cardiomyocytes. Interstitial and perivascular fibrosis of the myocardial tissue. Contraction band necrosis. Square nuclei. Myocell infiltration of leukocytes. Waviness	Haloperidol, Aripiprazole, Quetiapine, Promazine	Haloperidol, Aripiprazole, Quetiapine, Promazine
29	M	5	23	285	Ventricular chambers dilation	Myofiberbreak-up of cardiomyocytes. Interstitial and perivascular fibrosis of the myocardial tissue. Contraction band necrosis. Square nuclei. Waviness	Clozapine, Aripiprazole, Pimozide	Clozapine, Aripiprazole, Pimozide
30	M	9	19	435	No macroscopic alterations of the heart	Myofiberbreak-up of cardiomyocytes. Interstitial and perivascular fibrosis of the myocardial tissue. Contraction band necrosis. Square nuclei. Myocell infiltration of leukocytes. Waviness	Promazine	Promazine, Quetiapine
35	F	5	22	350	No macroscopic alterations of the heart	Myofiberbreak-up of cardiomyocytes. Interstitial and perivascular fibrosis of the myocardial tissue. Contraction band necrosis. Square nuclei. Waviness. Coronary atherosclerosis not hemodynamically significant (< 50%).	Clomipramine	Clomipramine
39	M	5	22.5	332	No macroscopic alterations of the heart	Myofiberbreak-up of cardiomyocytes. Interstitial and perivascular fibrosis of the myocardial tissue. Contraction band necrosis. Square nuclei. Waviness. Coronary atherosclerosis not hemodynamically significant (< 50%).	Clotiapine, Aripiprazole, Haloperidol	Clotiapine

## Discussion

SCD is often responsible for deaths in patients with schizophrenia treated with APDs, resulting in a reduced life expectancy and a risk that is four times higher than in the general population [19]. Most patients with schizophrenia are treated with APDs. In recent decades, there has been a shift from the use of typical APDs (e.g., thioridazine, haloperidol, chlorpromazine) to atypical APDs (e.g., clozapine, quetiapine, olanzapine). However, the relative contribution of each drug to the onset of cardiac side effects remains unclear. Overall, users of atypical APDs are at a higher risk, although no statistically significant difference has been observed [19]. Certain APDs can block ion channels in the ventricles, suppressing the rapidly activated delayed rectifier current (IKr), leading to QT prolongation and the development of serious arrhythmias [20]. A role for oxidative stress induced by APDs in the development of SCD and other cardiac damage has been hypothesized, though further evidence is needed to establish a causal relationship [21]. Serotonin receptors (5-HT receptors), particularly the 5-HT2A subtype, are present in cardiomyocytes and coronary blood vessels. Activation of these receptors can lead to vasoconstriction and, in some cases, myocardial hypertrophy and fibrosis, which align with our histological findings. Many atypical APDs, such as quetiapine and olanzapine, are potent 5-HT2A antagonists. While this action is primarily thought to be beneficial for psychiatric symptoms, it could also contribute to off-target cardiac effects [22]. Similarly, dopamine receptors, specifically the D1 and D2 subtypes, are also found in the myocardium. Dopamine plays a vital role in regulating cardiac contractility and blood pressure. The strong D2 antagonism characteristic of many first-generation APDs, such as haloperidol, and some atypical ones could disrupt this normal function, potentially contributing to arrhythmias and SCD [23–25].

A forensic study by Mehtonen et al. [26] conducted a 3-year retrospective analysis of patients treated with APDs who died from SCD and underwent autopsy; however, a comprehensive forensic description of the cadavers and heart tissue was not provided. The cardiac effects of clozapine were investigated by Khan et al. [27], who estimated a risk of SCD and myocarditis in clozapine users. Myocarditis was observed even 15 days after discontinuation of clozapine, highlighting the need for closer monitoring in patients on this drug.

Paratz et al. [28] found that people with schizophrenia had a significantly higher risk of sudden cardiac death (SCD) compared to the general population. The study, which was a large forensic series, revealed a frequent diagnosis of non-ischemic cardiomyopathies as the cause of death in these cases. Additionally, individuals with schizophrenia who experienced SCD were more likely to have cardiovascular risk factors like smoking and alcohol use, as well as to be on QTc-prolonging medications. While other studies [29–31] did not find specific genetic mutations clearly causing sudden death in patients with schizophrenia, they did identify shared genetic markers between certain arrhythmic disorders and schizophrenia.

In the present study, the effects of APDs on cardiac tissue were evaluated, minimizing the potential bias from other risk factors that are often present in these patients. Specifically, patients who died from SCD and had other concomitant risk factors (e.g., smoking, obesity, history of heart disease) were excluded. Additionally, patients under 18 years, who may have unrecognized genetic abnormalities contributing to SCD, and those over 39 years, were excluded to avoid age-related cardiac damage. Applying these criteria, only 7 cases were included, and these cadavers underwent an autopsy with a particular focus on cardiac tissue. The study population consisted of 6 males and 1 female, with ages ranging from 25 to 39 years (median age = 29 ± 4.71 SD). The duration of psychosis and treatment with APDs ranged from 5 to 10 years (median = 5 years). The body mass index (BMI) ranged from 19 to 23 (median = 22 ± 1.6 SD). None of the patients had cardiovascular risk factors or a history of substance abuse at the time of death.

Ray et al. [32], in a retrospective cohort study, calculated the incidence of SCD among current users of APDs, demonstrating that both typical and atypical APD users had higher rates of SCD compared to non-users, with an incidence-rate ratio of 1.14. This association was observed in a dose-dependent manner for both types of APDs. The authors concluded that current use of both typical and atypical APDs increases the risk of SCD in a dose-dependent fashion. Another complication associated with APD use and SCD is myocardial infarction. While the 1992 study by Kahn et al. [33] reported a 17-fold increased risk of myocardial infarction in patients using psychotropic drugs, it is important to consider its early context in this field. The study primarily focused on myocardial infarction, a finding supported by autopsy evidence of coronary occlusion. However, its conclusions regarding sudden cardiac death, particularly in women, may be limited given the time of publication and the authors’ subsequent research on other conditions.

Numerous post-mortem studies have analyzed the correlation between SCD and APD use, often utilizing hospital databases or national registries that include death certificates. However, as noted by D’Errico et al. [21], autopsy studies on this topic remain limited. Kelly et al. [34], compared autopsy data from individuals who died from SCD in Maryland between 1990 and 2004, focusing on patients with psychopathy treated with risperidone and clozapine. No significant differences were found between these two groups, though cardiac evaluation revealed that 30% of patients had atherosclerosis, fibrosis, or hypertrophy. Vang et al. [35] described two cases of patients treated with olanzapine who died from myocarditis, with histological and toxicological evidence of eosinophilic myocarditis. Ruschena et al. [28], in an epidemiological study conducted in New York’s psychiatric hospital, identified 22 deaths related to APD use, though no autopsy was performed. Of these, 15 were attributed to acute coronary syndrome (ACS), two to aortic dissection, two to myocarditis, and one to commotio cordis. Another study [36] examined over 7,000 patients in Maryland’s psychiatric hospital from 1989 to 2013, finding 57 deaths from SCD, 51 of which were autopsied. The causes of death were primarily myocardial infarction (52.9%), myocarditis (5.9%), and dilated cardiomyopathy. In this study, there was an almost equal ratio of males to females, and the patients had been diagnosed with schizophrenia for almost 27 years. All patients had received APDs within 24 h of death, and a large proportion had a history of cardiovascular disease with cigarette smoking. A retrospective study [37] examined 391 autopsies of schizophrenic patients from 2008 to 2012, of various races and ethnicities. Sudden death (SD) occurred in 64.2% of cases, 12% of deaths were due to accidents, 11.5% of deaths were due to suicide, and 9% were due to homicides, with the remainder of deaths being undetermined. In 80% of cases of SD, the cause was cardiac. Body mass index was highly variable, however, morphology, cardiac abnormalities, and forensic histopathological abnormalities were not clarified in this study. D’Errico et al. [21] emphasized the scarcity of autopsy studies on patients with psychopathy treated with APDs who die from SCD and called for more research in this area. Sweeting et al. [38] analyzed a database of schizophrenia patients treated with APDs who underwent autopsy, noting that 62% were overweight or obese and 23% died from vascular causes, suicide, or drug abuse. In 11% of cases, the cause of death was undetermined, and in patients under 40, cardiovascular death was more common. Additionally, 50% of patients were found to have used narcotic substances, which may explain the high incidence of deaths due to drug overdose [39].

In this study, the histological findings were compared with those in previously published research. According to various authors [40–43], the forensic diagnosis of SCD involves identifying coronary atherosclerosis/stenosis, myocardial fibrosis, myofiber break-up, interstitial and perivascular fibrosis, leukocyte or eosinophilic granulocyte infiltration, contraction band necrosis, and myocyte degeneration. Granulocytic infiltration is particularly important in the evaluation of clozapine-induced myocarditis and expands the potential range of drugs that could lead to a similar cardiotoxic response [44]. Myofiber break-up is a known marker of ventricular fibrillation, which often precedes SCD [45].

In the present study, the heart weight ranged from 332 g to 520 g (median = 350 ± 78 SD), which is considered normal based on the patients’ BMI [46, 47]. Macroscopic examination revealed no abnormalities in 3 cases, hemorrhagic petechiae and ischemic areas in the right ventricle in 2 cases, and left ventricular hypertrophy in 1 case. Microscopic examination revealed myofiber break-up, interstitial and perivascular fibrosis, contraction band necrosis, waviness, and square nuclei in all cases. Coronary atherosclerosis was present in 3 cases (< 50%), and leukocyte infiltration was observed in 2 cases.

These findings, when compared to existing literature, suggest that SCD was the likely cause of death in all cases. Histological examination showed signs of chronic myocardial injury, leading to arrhythmic SCD, and supports the conclusion that APDs contributed to this cardiac damage over time. While the histopathological findings described in our study—such as myocardial fibrosis, myofiber break-up, and contraction band necrosis—can also be observed in cases of sudden death in the general population without structural heart disease, the key difference lies in their preponderance and severity. In this study, which consists of young adults, the presence of such alterations is particularly significant. In the general population of the same age group, these findings are far less common and, when they are present, tend to be less extensive. Therefore, while the findings are not unique, our study highlights that in patients with schizophrenia on antipsychotic therapy, these alterations are present more prominently and in a population where one would not expect to find them with such frequency and intensity. This study reveals that antipsychotic drugs (APDs) may cause direct cardiac damage. Histological analysis of the deceased patients’ hearts showed structural changes, including myocardial fibrosis and contraction band necrosis.

Regarding toxicological analysis, APDs were found in both blood and hair in all cases, confirming that the patients had used APDs both shortly before and in the months leading up to death. In four cases, the same APDs were found in both blood and hair (Cases 1, 3, 4, and 6), while in the remaining three cases (Cases 2, 5, and 7), the drugs identified in hair differed from those found in blood. Blood toxicology frequently detected haloperidol, promazine, aripiprazole, and clozapine (in 3 cases), followed by quetiapine (2 cases), and clomipramine, paliperidone, and pimozide (1 case each). Hair toxicology most commonly detected quetiapine, clozapine, and promazine (3 cases), followed by haloperidol and aripiprazole (2 cases), and pimozide and clomipramine (1 case each). The discordance between the APDs found in blood and hair suggests that patients were undergoing polytherapy with APDs, with some drugs being used inconsistently in the months preceding death.

While the small sample size and polytherapy patterns limit the ability to identify specific APDs responsible for cardiac toxicity, this study is the first to conduct a targeted investigation of APDs in myocardial tissue with a detailed autopsy. This research provides valuable insight into how APDs can cause cardiac injury and SCD, even at therapeutic doses in otherwise healthy individuals with no history of heart disease or risk factors. Future studies should include genetic testing and molecular autopsies, which have proven effective in diagnosing SCD in cases with normal hearts, particularly with respect to genetic mutations in RyR2, KCNQ1, KCNH2, and SCN5A. However, the high costs of molecular autopsies remain a limiting factor [48]. Furthermore, only one study [49] identified a genetic variant associated with SCD in APDs users, specifically the nonsynonymous single nucleotide polymorphism rs10503929 in the neuregulin 1 gene (NRG1).

## Conclusion

SCD due to APDs is an emerging concern, particularly given the rising incidence of schizophrenia among younger patients, which is also partly attributed to increased awareness and diagnosis of the disorder. However, the use of these medications exposes individuals to an elevated risk of SCD, accompanied by cardiac morphological changes. This factor must be carefully considered when prescribing APDs. The present study highlights the significant structural alterations in the heart caused by these drugs, which may lead to SCD in otherwise healthy young adults with no history of heart disease, even when taken at therapeutic doses. Future research could incorporate molecular autopsies in schizophrenia patients treated with APDs who have died from SCD. Such studies would be valuable for evaluating the role of genetic factors in these individuals’ susceptibility to SCD [50, 51]. The results of this study underscore the critical need for more rigorous cardiac monitoring in schizophrenic patients treated with APDs, even in the absence of traditional cardiovascular risk factors. This proactive approach is essential for preventing SCD.

## Limitations of the study

This study has several limitations. The first is the limited sample size, primarily due to the stringent inclusion criteria, which, while ensuring greater homogeneity, also led to a reduction in the number of cases. Another limitation is the inclusion of only one female participant. Although this finding is random, it is also consistent with epidemiological data suggesting a higher incidence of schizophrenia in men compared to women [52]. However, the inclusion of only one female participant may limit the generalizability of the results to the general population. Finally, genetic analysis was not performed on the patients at the time of autopsy due to the high cost of such analyses. Conducting genetic analyses would have further contributed to the research by evaluating potential genetic predispositions to sudden cardiac death (SCD). Moreover, the sample size and polytherapy patterns of patients do not allow for an assessment of the individual cardiotoxicity of APDs. Future studies incorporating genetic evaluation could provide valuable insights into this aspect. The absence of specialized stains like Masson’s trichrome is a recognized weakness, as it limited our ability to provide a more definitive and quantitative assessment of the observed myocardial fibrosis. We recommend that future studies utilize a broader range of staining techniques to more thoroughly evaluate potential cardiac pathologies in this patient population. Future studies should include histological examination of the conduction system.

## Key points

1. Antipsychotic drugs therapy is significantly associated with an elevated risk of sudden cardiac death, particularly among young adult patients diagnosed with schizophrenia.

2. Post-mortem analyses revealed that antipsychotic drugs can induce profound structural cardiac changes (e.g., myocardial fibrosis, myofiber disruption) that are consistent with an arrhythmogenic mechanism for sudden cardiac death.

3. Future investigations should incorporate molecular autopsy and genetic analysis to elucidate genetic predispositions influencing susceptibility to antipsychotic drugs induced sudden cardiac death.

## Data Availability

All data are included in the main text.
